# Bacteriology in the Treatment of Disease

**Published:** 1899-11

**Authors:** C. T. Whinery


ARTICLE III.

### BACTERIOLOGY IN THE TREATMENT OF DISEASE.

C. T. WHINERY, D. D. SC.

**Read before the Toledo Dental Society.**

The discovery of bacteria and their association with different diseases has opened up a new epoch in the history of the dental and medical professions. The honors have not been entirely with the medical men, for, while Koch, Pasteur, Frankel and Sternberg have been working in their specialties, Miller, Boedecker, Blank and others have been revealing to us the hidden science of our profession. It is hard to believe that there is a physician or dentist living to-day who would doubt the existence of such a science or who would be so conservative in his practice as not to apply it more or less in his daily work. Yet instances come up quite frequently where both physicians and dentists practice entirely without any regard whatever to bacteriology.

To verify this statement I will relate a case that came under our observation. About two years ago one of the local physicians (enjoying the luxury of a good home and the benefits of a lucrative practice), brought a girl about fifteen years of age, afflicted with necrosis of the frontal bone, to the office. After the bandage and cotton were removed, the case showed where the disease had destroyed the overlying tissues and the bone was exposed to the size of a twenty-five cent piece, with but a thin lamina remaining to cover the brain. There was pus present in an appreciable amount along the entire border of the affected part. Granulation tissue was showing itself in some places, but with the lack of antiseptic conditions it had no chance whatever to become healthy and work toward restoration. The object of the physician's visit was to use a large bur in the dental engine and cut away the diseased bone. This was done without sterilizing or even washing his hands or the bur. The grindings were blown from the cavity with his mouth, and any remaining pieces were wiped out with the bandage that the patient had worn. After the operation was completed the same cotton still moist with pus was put into the wound again, where each germ could form a new colony of its own and tear down as fast as granulation took place. When asked if he ever used any antiseptic dressing or precaution in some cases, he merely smiled and said he did not believe in such trash and had never carried it out in his practice. The case no doubt would have been hard enough to treat under the most favorable conditions, but that does not excuse the operator rom employing the best methods that the profession offers, neither does it apologize for his thick skin and bull-headedness. We did not see the case again and cannot say what was the ultimate result. But it is safe to assume that the disease passed through the lamina of bone that protected the brain, and that this organ became affected also.

In the treatment of diseases of bacterial origin, the sooner we render conditions adverse to the proliferation and welfare of the germ, the sooner will we reach the desired results. Germs thrive and multiply under definite temperature (in the body that temperature is 98.4 degrees), with slight variation to some and greater to others. The germ of typhoid fever, for instance, has a wide range of temperature. It is said that it can hibernate in a cake of ice and then come out like the ground-hog in the spring, full of life and vigor to enter upon its coming campaign. Germs with such resisting powers do not pass through these periods without first protecting themselves. In order to bring about this fortification, they inwardly collect their vital principles into a granular mass, which is surrounded by a tough resisting membrane impervious to temperature and moisture.

All germs require moisture, some need oxygen, others do not, and all demand plenty of food, and that means more than three meals a day. In the mouth we find every requisite for the flourishing of a colony of bacteria. There is the proper temperature and sufficient moisture (and I suppose among those requiring moisture could be classed the germs, which belong to the toper gang, who never see a sober moment, and look upon water as a thing to bathe in and not fit to drink). Then there is a constant supply of oxygen for those that require it and food in abundance.

There are comparatively few anærobic bacteria, those which are able to exist without the presence of air. But among this class are the ones which produce decay under air-tight fillings. Every dentist has noticed its ravages in such cases to a greater or less degree. It is, therefore, of highest importance to thoroughly remove all decay before filling. It becomes necessary, however, to make one exception to this last statement, and only one. There are times when a thorough excavation of decay would expose a live pulp. Soft decay of course should always be removed, but the hard, brown layer so often found over pulps can be successfully sterilized with the many agents we have at command, and afterwards dehydrated with alcohol as a secondary precaution to prevent further activity of the germs in the tubules.

It follows that if infectious varieties find abode in the mouth, they are liable to assert their action if an opportunity is offered and so cause the mouth to become a center of infection.

What has the deposits of lime upon the teeth to do with the subject of this paper? Let us trace the effect of the deposits upon the tissues and I think we will readily see. As was said before, the mouth furnishes every requisite for the flourishing of a colony of bacteria, and also that the mouth should be in the best possible condition to fight its daily battles. The lime salts by their constant accumulation act as a continual irritant to the gum tissues and the gums use every means to hold their position, but the enemy are too strong for them and they are forced back. This allows room for more deposit and the same thing happens again. The distribution of lime, where there is a tendency to accumulate at all, is usually general and the irritation then becomes general, followed by a feverish and swollen condition of the gums, with the ultimate breaking down of their substance and adjacent parts and rendering them unable to resist the action of the germs present in the mouth; if conditions are favorable a well-developed case of pyorrhea follows. The presence of the lime deposits thus acting as the chief exciting cause lowers the vitality of the gums to the point of bacterial infection.

Of the many kinds of bacteria that are always present in the mouth, there is only a small per cent. of them that are capable of doing much damage. But among this few will sometimes be found the germs of typhoid fever, the bacillus of tuberculosis and diphtheria and others of such severe type. Beside these there may be mentioned the fermentative variety which act upon the food remaining after a meal, forming acids which attack the lime salts of the teeth, producing the so-called dental caries. The saliva being neutral or slightly alkaline in reaction gives nature no remedy to overcome the action of these germs. It therefore becomes the dentist's duty to use and prescribe such washes as will help nature to exterminate these pests, and to teach the patient with regard to proper care of the teeth in order to keep the mouth in as healthy a condition as is possible.

In the foregoing I have not attempted to cover the subject thoroughly, but merely to emphasize a few of the important principles necessary in the treatment of diseases of bacterial origin.—*Indiana Dental Journal.*

